# An Equation to Estimate the Concentration of Serum Apolipoprotein B

**DOI:** 10.1371/journal.pone.0051607

**Published:** 2012-12-19

**Authors:** You-Cheol Hwang, Hong-Yup Ahn, Woo Je Lee, Cheol-Young Park, Sung-Woo Park

**Affiliations:** 1 Division of Endocrinology and Metabolism, Department of Medicine, Kyung Hee University School of Medicine, Kyung Hee University Hospital at Gangdong, Seoul, Korea; 2 Department of Statistics, Dongguk University-Seoul, Seoul, Korea; 3 Department of Internal Medicine, Asan Medical Center, University of Ulsan College of Medicine, Seoul, Republic of Korea; 4 Division of Endocrinology and Metabolism, Department of Internal Medicine, Sungkyunkwan University School of Medicine, Kangbuk Samsung Hospital, Seoul, Korea; University of Milan, Italy

## Abstract

**Background:**

Several large prospective studies have demonstrated that apolipoprotein B (apoB) has greater value in predicting cardiovascular risk than any other lipid measurements. Currently, however, serum apoB levels are not routinely measured, because of the additional cost. The aim of this study was to develop an equation to estimate apoB from conventional lipid measurements including total cholesterol, HDL cholesterol, and triglycerides.

**Methods:**

Data from a total of 78,127 subjects (47,057 men and 31,070 women), aged 15 to 88 years (mean age 41.8 years) were reviewed to develop an apoB equation. Additional datasets from the same institution and the NHANES obtained in 2007–2008 were used for internal (n = 73,445) and external validation (n = 3,097), respectively.

**Results:**

We developed an apoB equation based on a linear regression model that contains total cholesterol, triglycerides, and HDL cholesterol as terms (model 1). To more precisely estimate the serum apoB level, we adjusted mode1 1 using a cutoff serum triglyceride value of 270 mg/dl (model 2). Model 2 showed more randomly distributed residuals in patients with diabetes, atherogenic dyslipidemia, and those taking lipid-lowering agents than model 1. The residuals in the development, internal validation, and external validation datasets were also randomly distributed around 0 with no clear trends.

**Conclusion:**

The new equation we developed to estimate serum apoB concentrations is accurate and can be used in diverse subgroups of patients including those with diabetes, atherogenic dyslipidemia, and those taking lipid-lowering agents.

## Introduction

Apolipoprotein B (apoB) is a major structural protein for atherogenic lipoproteins including chylomicron, VLDL, intermediate-density lipoprotein, large buoyant LDL, and small dense LDL. ApoB is required to transport lipids from the liver and gut to peripheral tissues. In general, one molecule of apoB is present on each lipoprotein particle; therefore, the total apoB level represents the total number of atherogenic particles and reflects the atherogenic potential of the whole lipoprotein fraction [1].

Several large prospective studies have demonstrated that the apoB level is a better predictor of cardiovascular risk than any other lipid measurements [2]–[4]. In the AMORIS study, apoB was found to be superior to LDL cholesterol as a marker to assess cardiovascular risk, particularly in patients with normal or low LDL cholesterol levels [2]. Data from numerous clinical trials with statins have also reported that residual risk is more strongly associated with the apoB level rather than LDL or non-HDL cholesterol levels [5], [6]. In the THROMBO study in patients who had recovered from myocardial infarction, higher apoB levels were independently associated with an increased risk of recurrent events, whereas conventional lipid measurements were not [7].

However, despite the clinical benefits of apoB levels over conventional lipid measurements for the assessment for cardiovascular risk, apoB levels are not routinely measured because of the additional cost. Therefore, we developed an equation to estimate the serum apoB concentration based on the conventional lipid measurements included in Friedewald's formula [8]. We validated the performance of our equation using both an internal and external dataset and applied the equation to diverse subgroups of patients to test its validity.

## Methods

### Ethics statement

No specific informed consent was obtained. The requirement for written or verbal informed consent was waived by the Institutional Review Board during the planning phase of this study because researchers were only allowed to assess the database for analysis purposes, and the database did not contain any personal identifying information.

Ethics approval for the study protocol and data analysis was obtained from the Institutional Review Board of Kangbuk Samsung Hospital, and the protocols used in this study complied with the Declaration of Helsinki.

### Dataset for equation development

Study participants were recruited from subjects who visited the Health Screening Center at Kangbuk Samsung Hospital for a routine medical check-up between January 2009 and December 2009. During that period, a total of 98,248 subjects received a medical check-up. Among them, 78,127 subjects (47,057 men and 31,070 women), aged 15 to 88 years (mean age 41.8 years), who had conventional lipid measurements including total cholesterol, triglycerides, HDL cholesterol, and directly measured LDL cholesterol and serum apoB levels, were enrolled in the study.

In this study, diabetes mellitus was defined by the presence of one of the following: (1) fasting glucose level ≥126 mg/dl; (2) taking oral hypoglycemic agents; (3) a self-reported history of diabetes; or (4) HbA1c level ≥6.5% [9]. In addition, atherogenic dyslipidemia was defined by both elevated triglyceride (≥150 mg/dl) and reduced HDL cholesterol levels (<40 mg/dl in men or <50 mg/dl in women) [10].

### Dataset for internal validation

To validate how accurate our estimated apoB levels were based on the 2009 dataset, we used another dataset from the same institution from January 2008 and December 2008. During that period, a total of 94,027 subjects received a medical check-up and among them, 73,445 subjects (44,341 men and 29,104 women), aged 18 to 87 years (mean age 41.7 years) with lipid parameters identical to patients included in the 2009 dataset were used for internal validation.

### Dataset for external validation, NHANES 2007–2008

We also validated how accurate our estimated apoB levels were using data from NHANES 2007–2008. During that period, a total of 3,097 subjects (1,566 men and 1,531 women, mean age 43.6 years) with measured total cholesterol, triglyceride, HDL cholesterol, and apoB levels, were enrolled in the analysis. Data analyses were performed using special sampling weights.

### Clinical and laboratory data for equation development and internal validation

At baseline, a complete physical examination of each subject was performed, and a personal medical history, family history of metabolic diseases, and lifestyle factors were determined using a standardized questionnaire.

All blood samples were obtained in the morning following an overnight fast of 12 to 14 hours. Serum glucose was measured by the hexokinase method using an autoanalyzer (Advia 1800; Siemens, Berlin, Germany). The inter-assay coefficient of variation (CV) was 1.0%. Serum total cholesterol, triglyceride, HDL cholesterol, and LDL cholesterol levels were determined using the autoanalyzer. Serum apoB concentrations were determined by the immunoturbidometric method (Advia 2400 autoanalyzer; Siemens, Berlin, Germany) with inter-assay CVs ranging from 2.9 to 4.7%. HsCRP was analyzed by particle-enhanced immunonephelometry with the BNIITM System (Dade Behring, Marburg, Germany) using a lower detection limit of 0.175 mg/l. Our clinical laboratory participates in annual inspections and surveys by the Korean Association of Quality Assurance for Clinical Laboratories, and has been accredited for quality control and performance of various measurements.

### Clinical and laboratory examination of NHANES 2007–2008

Serum total cholesterol, triglyceride, and HDL cholesterol levels were determined by the enzymatic method using a Roche Modular P chemistry analyzer (Roche Diagnostics, 9115 Hague Road, Indianapolis, IN 46250). Serum apoB concentrations were determined by the Dade-Behring BN ProSpec nepholometric immunoassay with a CV of 2.0–6.2%.

### Statistical methods

To develop an apoB equation, we examined various linear regression models that included conventional lipid measurements such as total cholesterol, triglyceride, and HDL cholesterol levels as terms. In addition, taking into account that the estimated LDL cholesterol is valid only for a limited range of triglycerides, we adjusted the regression coefficients in the final model according to a cutoff value of triglycerides. To determine the triglyceride cutoff value, we used the Bayesian information criterion (BIC). We used two additional datasets for internal and external validation of the apoB equation. For the internal dataset, errors between directly measured apoB and estimated apoB levels were plotted as residual plots. Regression coefficients estimated for the internal dataset were compared with those estimated using the apoB equation. For the external dataset, the same methodology was followed. To determine the performance of the apoB equation in various subgroups of patients, we plotted locally weighted scatter-plot smoothing (LOWESS) curves. Statistical analyses were performed with R version 2.9.2 (http://www.r-project.org). All statistical tests were two-sided and *P*<0.05 was set as the statistical significance level.

## Results

### Demographic and clinical characteristics

The clinical characteristics of the study participants in each dataset are shown in Table 1. The mean age of patients in the development dataset was 41.8 years, and 39.8% of the participants were female. Patients in the internal validation data had similar clinical characteristics to those in the development dataset (there were not significant differences in measured parameters between the two datasets). However, subjects in the external validation dataset were older and more likely to have low apoB levels than those in the development and internal validation datasets.

**Table 1:** Participants' characteristics.

	Development dataset (n = 78,127)	Internal validation dataset (n = 73,445)	External validation dataset (n = 3,097, population = 111,678,346)
Age (years)	41.8 (8.5), 35–46	41.7 (8.4), 36–46	43.6 (18.7), 27–63
Female (%)	31,070 (39.8)	29,104 (39.6)	1,531 (50.8)
Body mass index (kg/m^2^)	23.6 (3.2), 21.3–25.5	23.6 (3.1), 21.4–25.6	27.7 (6.3), 23.4–31.5
Total cholesterol (mg/dL)	195.6 (33.7), 172–217	195.2 (33.8), 172–216	191.3 (41.5), 159–216
Triglycerides (mg/dL)	127.0 (85.4), 74–154	127.5 (86.7), 73–156	130.3 (98.0), 73–158
HDL cholesterol (mg/dL)	55.6 (13.0), 46–63	55.2 (12.7), 46–63	53.3 (15.2), 43–62
LDL cholesterol (mg/dL)	113.1 (30.0), 92–132	110.7 (29.4), 90–129	NA
Calculated LDL cholesterol (mg/dL)	114.6 (30.1), 93.8–133.4	114.5 (30.4), 93.8–133.4	112.6 (35.0), 85–132
Apolipoprotein B (mg/dL)	96.4 (22.9), 79.8–110.9	97.5 (24.0), 80.4–113.0	90.5 (24.8), 71–106
Estimated apolipoprotein B (mg/dL)	96.2 (22.2), 80.2–110.6	96.2 (22.3), 80.2–110.5	93.6 (26.3), 73.3–109.6

Data are expressed as means (SD), inter-quartile ranges or frequencies (%).

NA, non-available.

The NHANES data analyses were performed using special sampling weights.

To convert serum cholesterol levels to millimoles per liter, multiply by 0.0259. To convert the serum triglyceride level to millimoles per liter, multiply by 0.0113. To convert the serum apolipoprotein B level to grams per liter, multiply by 0.01.

### Development of an equation to estimate apoB levels

**Figure 1:**
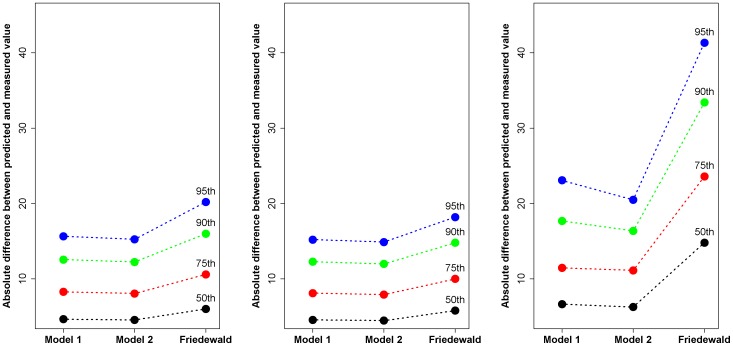
Performance of apoB equations and Friedewald's formula for ranges of triglyceride levels. All subjects (**A**), subjects with triglycerides <270 mg/dl (**B**), and subjects with triglycerides >270 mg/dl (**C**). The 50, 75, 90, or 95% of subjects had estimation errors less than the Y-axis values (mg/dl). The closer the percentiles of errors to 0, the better the corresponding model.

First, we developed a simple apoB equation model (model 1), as shown below:
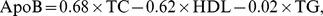
which included serum total cholesterol (TC), HDL cholesterol (HDL) and triglyceride (TG) levels, as in Friedewald's equation to estimate LDL cholesterol levels. Similar to Friedewald's equation, which is valid only for a limited range of triglycerides, we found that the simple apoB equation model performed well only for a certain range of triglycerides. To extend the valid range of the apoB equation, we considered a more complex model with a structure essentially identical to that of model 1 but with regression coefficients adjusted by the cutoff value of triglycerides (model 2), as follows:
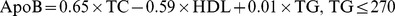


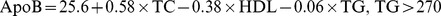
In this equation, the regression coefficients of serum total cholesterol, HDL cholesterol, and triglyceride levels are dependent on the triglyceride cutoff value. We found that a serum triglyceride cutoff value of 270 mg/dl had the lowest BIC score (Fig. S1). We compared the performance of the two models at predicting the directly measured serum apoB level using the development dataset. The improvement in performance obtained by incorporating a serum triglyceride cutoff value was also evaluated by comparing the percentage absolute differences between the estimated and directly measured serum apoB levels between the two models. A lower percentage absolute difference indicates a narrower distribution of estimated serum apoB levels, which in turn reflects more accurate estimation of the directly measured serum apoB levels. Three plots of absolute differences are presented for all subjects, those with triglycerides <270 mg/dl, and those with triglycerides >270 mg/dl. No difference in performance was noted between the two models below the triglyceride level of 270 mg/dl; however, model 2 (the final model) provided a more precise estimate of directly measured serum apoB levels than model 1 for patients with triglyceride levels higher than 270 mg/dl, especially for the 90^th^ and 95^th^ percentiles of the percentage absolute differences (Fig. 1). In other words, our final model performed well for all ranges of triglyceride levels. Therefore, we used model 2 from this point onwards. In addition, we compared the performance of our equation with the established model that is currently used to estimate the serum LDL cholesterol, namely Friedewald's formula. Our equation more precisely estimated directly measured serum apoB concentrations than Friedewald's formula estimated the serum LDL cholesterol level, especially in subjects with high triglyceride levels (>270 mg/dl) (Fig. 1).

### Performance comparison

**Figure 2:**
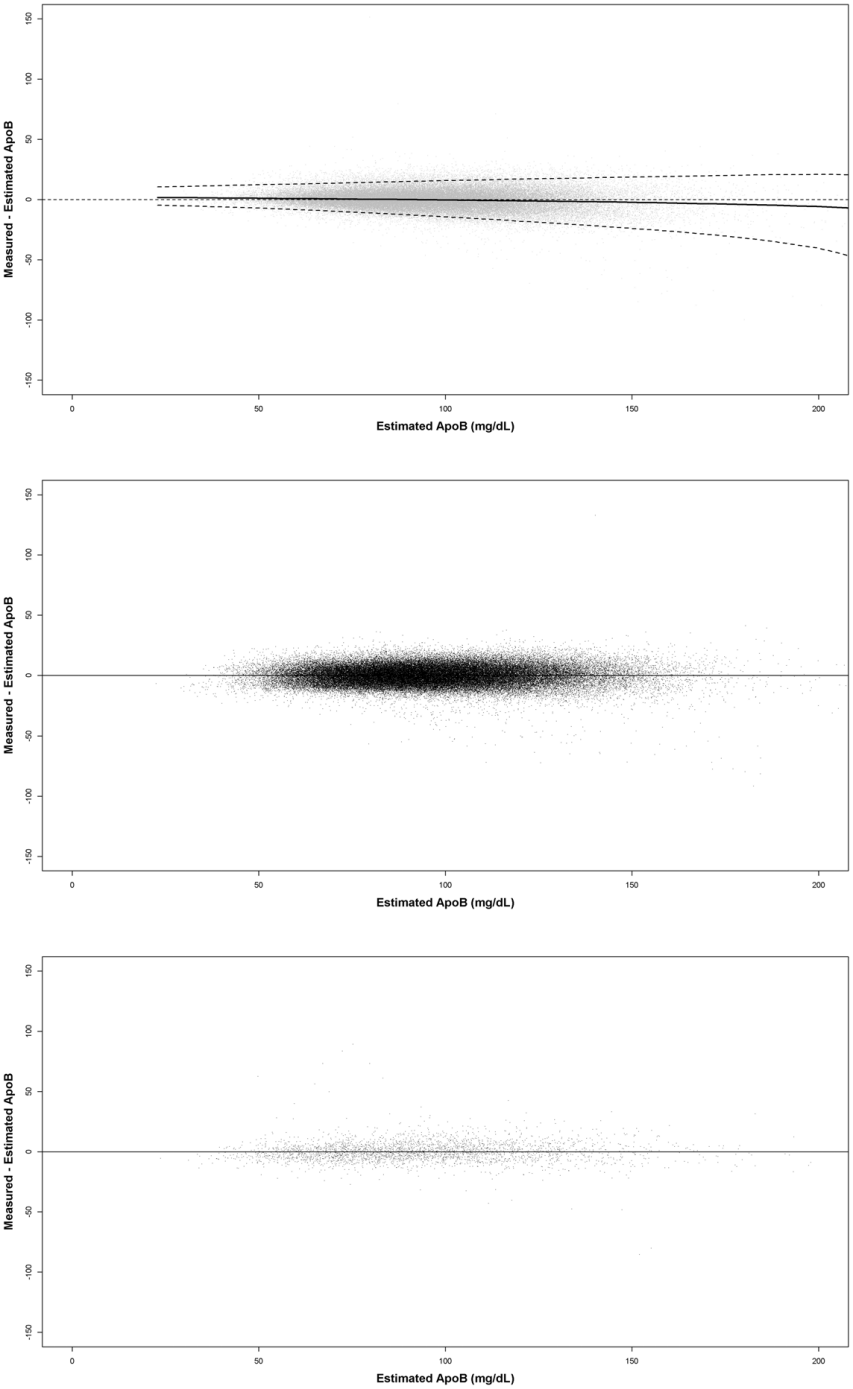
Residual plots for the datasets. Development dataset (**A**), internal validation dataset (**B**), and external validation dataset (**C**). Solid line is the 50^th^ quantile regression line, and the dotted lines are the 2.5^th^ and 97.5^th^ quantile regression lines, respectively.

To illustrate the performance of the apoB equation in the development, internal validation, and external validation datasets, we constructed residual plots i.e. plots of the difference between the measured serum apoB level and the estimated apoB level versus the estimated apoB level, where the apoB level was estimated using our apoB equation (Figs. 2A–2C). In Figure 1A, which is based on the development dataset, the residuals are randomly distributed around 0. The solid line indicates the 50^th^ percentile and the dashed lines indicate the 2.5^th^ and 97.5^th^ percentiles, respectively. For the internal validation dataset, the residuals were also randomly distributed around 0 and no trend was observed. For the external validation dataset, no trend was observed and the residuals were randomly distributed. Therefore, the estimated apoB predicted the directly measured apoB without bias.

### Performance of the apoB equation in patient subgroups

**Figure 3:**
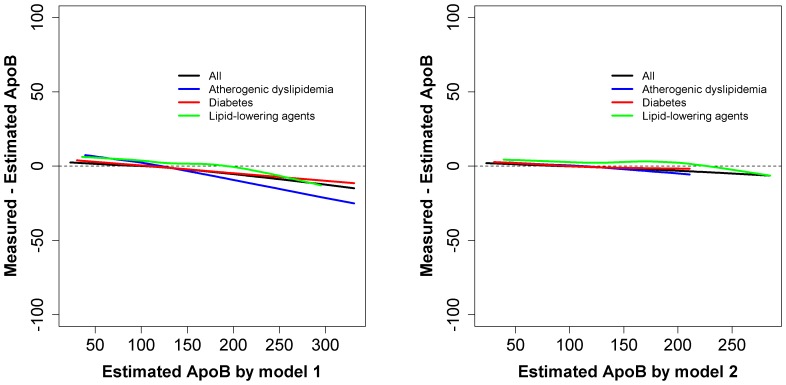
Comparison of the two apoB equations for subgroups. All subjects (black), those with atherogenic dyslipidemia (blue), diabetes (red), or those taking lipid-lowering agents (green) (**A**, model 1 and **B**, model 2). The lines are LOWESS curves of errors ( = measured - estimated apoB); the closer the curve to 0, the better the fit.

We determined the performance of the apoB equation (model 2) in various subgroups of subjects stratified according to age, body mass index (BMI), systolic blood pressure, fasting glucose, glycated hemoglobin tertiles, or gender. Although some differences were observed according to tertiles based on the various groups and gender, the LOWESS curves were around 0 for these tertiles (Fig. S2). The LOWESS curves of the differences between the measured serum apoB and the estimated apoB for all subjects, patients with diabetes (n = 9,084, 11.6%), patients with atherogenic dyslipidemia (n = 4,763, 6.1%), and patients taking lipid-lowering agents (n = 2,650, 3.4%) are shown in Figs. 3A and 3B. LOWESS curves were supposed to be around 0. LOWESS curves were supposed to be around 0. When model 1 was used, a decreasing trend was seen for all subgroups, thus suggesting overestimation of apoB levels. However, when model 2 was used, no such decreasing trend was observed. These results indicate that the apoB equation (model 2) can be used both in healthy subjects and in patients with diabetes, atherogenic dyslipidemia, and those taking lipid-lowering agents.

## Discussion

Numerous studies have demonstrated an association between serum apoB levels and residual cardiovascular risk. Recently, a consensus panel convened by the American Diabetes Association and the American College of Cardiology recommended a greater focus on non-HDL cholesterol and apoB levels in patients who are likely to have small LDL particles, such as diabetic patients. The consensus panel suggested that for statin-treated patients in whom the LDL cholesterol goal is <70 mg/dl (non-HDL cholesterol <100 mg/dl), apoB should be measured and treated to <80 mg/dl. For patients on statins with an LDL cholesterol goal of <100 mg/dl (non-HDL cholesterol <130 mg/dl), apoB should be measured and treated to <90 mg/dl [11]. In accordance, the task Force for the management of dyslipidaemias of the European Society of Cardiology and the European Atherosclerosis Society proposed that other factors, including serum apoB levels, should be investigated in middle-aged subjects with moderate cardiovascular risk, to precisely assess the real cardiovascular risk [12]. In particular, the task force team suggested that the apoB level may provide a better estimate of the concentration of atherogenic particles, especially in high risk patients with diabetes or metabolic syndrome, than other lipid markers. Therefore, if apoB measurement is available, target apoB levels should be <80 mg/dL and <100 mg/dL in patients at very high and high total cardiovascular risk, respectively [13].

In the present study, we developed an equation to estimate serum apoB concentrations using a database of subjects who visited the Health Screening Center at Kangbuk Samsung Hospital for a routine medical check-up. Approximately, 100,000 subjects receive an annual medical check-up at our institution, and many of these subjects are employees who are mandated by the Industrial Safety and Health Law in Korea to participate in annual or biannual health examinations. Therefore, although our data is not derived from a nationally representative population, we are confident that our results are representative of the characteristics of the general population of South Korea.

In the present study, we developed the apoB equation using conventional lipid measurements (total cholesterol, triglyceride, and HDL cholesterol levels) that are also used in Friedewald's equation to estimate LDL cholesterol [8]. Serum LDL cholesterol concentrations estimated using Friedewald's equation are widely accepted and used to estimate the risk of CVD. However, Friedewald's equation has some limitations [14]. First, unacceptable errors between estimated LDL cholesterol and directly measured LDL cholesterol increase progressively as plasma triglyceride levels increase. In the Framingham offspring study, when plasma triglycerides were <200 mg/dl, 85% of the estimated LDL cholesterol values were within 10% of the LDL cholesterol value determined by beta quantitation. However, this decreased to 77% and 59% when plasma triglycerides were 201–300 mg/dl and 301–400 mg/dl, respectively [15]. Similarly, we found limited associations between apoB and total cholesterol, HDL cholesterol and, especially, triglyceride levels as plasma triglyceride levels increased. Therefore, we incorporated a triglyceride cutoff value into our original apoB equation (which contained terms for total cholesterol, HDL cholesterol and triglycerides) to adjust for this finding, yielding model 2. We found that the correlation coefficient (95% CI) between the measured and estimated apoB levels was 0.938 when model 1 was used, and 0.945 when model 2 was used. The difference between the measured and estimated apoB level was less than 16 mg/dl in both models for 95% of all subjects; that is, only a 5% of our study subjects showed an apoB estimation error greater than 16 mg/dl. However, in subjects with triglyceride levels over 270 mg/dl, the 95^th^ percentile of the difference by model 1 was more than 24 mg/dl, whereas in model 2, it was about 20 mg/dl. In other words, model 2 performed better than model 1. In addition, we compared the performance of our equation with Friedewald's formula. The 75^th^ and 95^th^ percentiles of estimation errors in LDL cholesterol values in subjects with high triglyceride levels were more than 20 mg/dl and 40 mg/dl, respectively, when Friedewald's formula was used. In other words, application of Friedewald's formula resulted in an estimation error of more than 20 mg/dl in up to 25% of the study subjects. Moreover, the estimation error increased up to more than 40 mg/dl in 5% of the study subjects. Therefore, although some estimation errors may exist in our apoB equation, the equation effectively estimates directly-measured serum apoB concentrations in most cases, and the error was relatively small compared to that of Friedewald's formula (Fig. 1).

We used LOWESS curves to compare the performance of model 1 and 2 in subgroups of patients. As expected, model 2 did not over- or under-estimate apoB levels when all subjects were considered, whereas model 1 did. Although the coefficients of determination of model 1 and model 2 were as large as *R^2^* = 0.993 and *R^2^* = 0.994, respectively, we chose model 2 as our final model because the residuals of model 1 showed a decreasing trend. In model 2, for patients with diabetes and atherogenic dyslipidemia, apoB levels were slightly over-estimated, as the estimated apoB was more than 150 mg/dl, whereas apoB levels were under-estimated in patients taking lipid-lowering agents. It should be noted that the sample size for the subgroup taking lipid-lowering agents was only 2,650 (3.4%), indicating that it might be worthwhile to develop an apoB equation for this subgroup of patients based on a larger samples of patients taking lipid-lowering agents. However, the mean estimation error was 2.6 mg/dL (range, −6.3 to 4.4 mg/dL), which may be acceptable in clinical practice (Fig. 3). Similarly, although there were some differences between measured and estimated serum apoB levels according to age, BMI, fasting glucose, glycated hemoglobin, and systolic blood pressure tertiles, as well as gender, the differences were relatively small and are acceptable based on the LOWESS curves (Fig. S2). We next examined the performance of our equation (model 2) according to serum total and LDL cholesterol levels. We found that the mean estimation errors were less than 2 mg/dL. Therefore, the effects of serum total and LDL cholesterol on the accuracy of the equation appear to be minimal (data not shown).

Our present study has some limitations. First, although we used a large sample size to develop the equation, our equation development sample was not representative of the general population, and may have been biased towards healthier subjects. Second, we did not validate the clinical usefulness of the estimated apoB level with a prospective longitudinal outcome study. Therefore, it is uncertain whether the serum apoB concentration estimated using our equation would detect high-risk subjects more easily or earlier than conventional lipid measurements and directly-measured serum apoB levels in a clinical setting.

In summary, we developed an equation to estimate the serum apoB concentration based on total cholesterol, HDL cholesterol, and triglyceride levels. Our equation was accurate for the entire study population and for several subgroups, including diabetic patients, those with atherogenic dyslipidemia, and those taking lipid-lowering agents, and can be applied regardless of serum triglyceride levels.

## Supporting Information

Figure S1 **The goodness of fit by cutoff values of triglycerides.** The dotted vertical line is 270 (mg/dl) of triglycerides. The smaller BIC (Bayseian information criterion) is, the better the model is.
(TIF)

Figure S2 **Performance of the apoB equation (model 2) in subgroups.** The lines are LOWESS curves of errors ( = measured - estimated apoB); the closer the curve to 0, the better the fit.
(TIF)
