# Supplementary figures and images for: An Equation to Estimate the Concentration of Serum Apolipoprotein B
Source: PLoS One. 2012 Dec 19;7(12):e51607. doi: 10.1371/journal.pone.0051607 (PMC3526603; doi:10.1371/journal.pone.0051607)

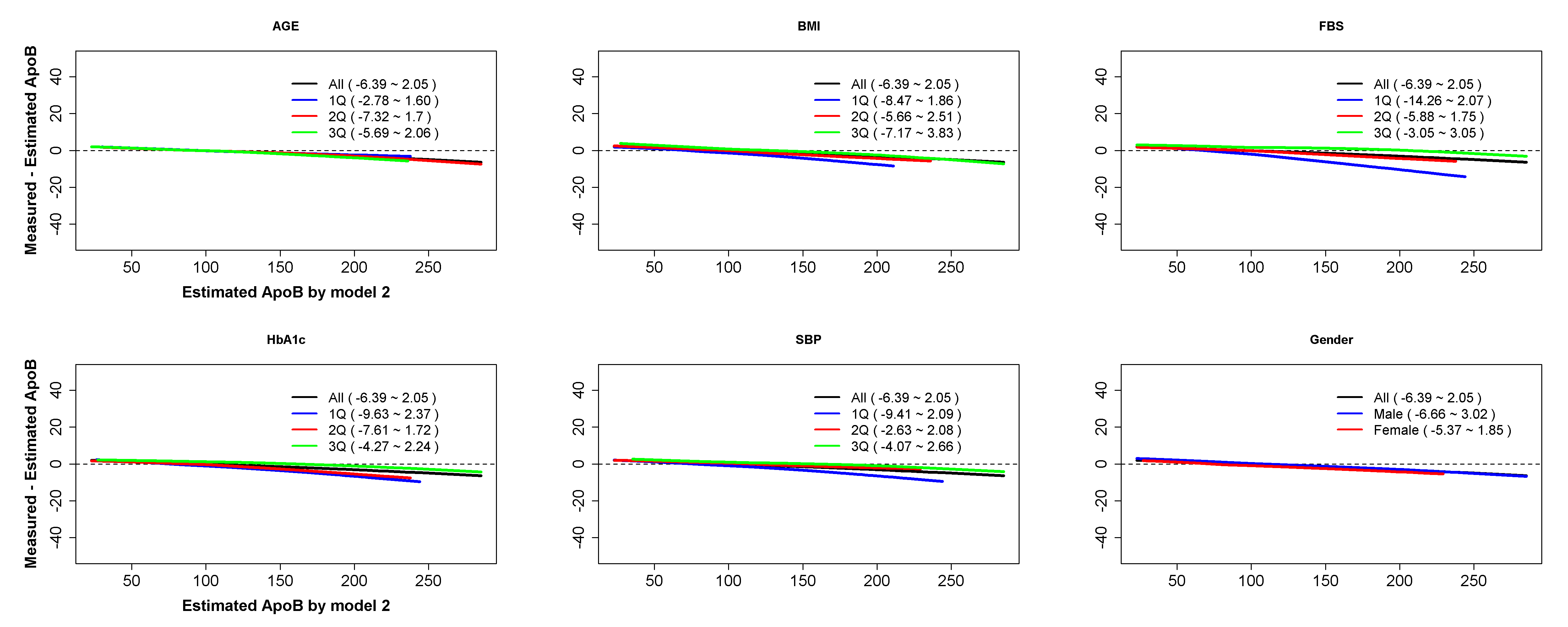

Supplement: Figure S2 — Performance of the apoB equation (model 2) in subgroups. The lines are LOWESS curves of errors ( = measured - estimated apoB); the closer the curve to 0, the better the fit. (TIF) [file pone.0051607.s002.tif]
